# The Impact of a Single Supervised Exercise Session in the Third Trimester of Pregnancy on the Physical Activity Levels of Pregnant Women—A Pilot Study

**DOI:** 10.3390/clinpract13050110

**Published:** 2023-10-03

**Authors:** Christos Chatzakis, George Mastorakos, Eleftheria Demertzidou, Anatoli Theodoridou, Konstantinos Dinas, Alexandros Sotiriadis

**Affiliations:** 1 Second Department of Obstetrics and Gynecology, School of Medicine, Aristotle University of Thessaloniki, 56403 Thessaloniki, Greece; cchatzakis@gmail.com (C.C.);; 2 Endocrine Unit of Aretaieion Hospital, Medical School, National and Kapodistrian University of Athens, 11528 Athens, Greece; 3 Midwifery Department, School of Health Sciences, International Hellenic University, 65404 Thessaloniki, Greece

**Keywords:** pregnancy, physical activity, supervised exercise session, medical personnel

## Abstract

Background: Despite the numerous beneficial effects of physical exercise during pregnancy, the levels of physical activity remain low. The aim of the study is to investigate the impact of a single supervised physical exercise session on the overall physical activity levels of pregnant women. Methods: During the third trimester, pregnant women attending our outpatient clinic were requested to assess their physical activity levels using the International Physical Activity Questionnaire (IPAQ). Additionally, they were invited to participate in a supervised 30 min mild–moderate-intensity aerobic exercise session (stationary bike ergometer) under the guidance of medical personnel. Subsequently, physical activity levels were reevaluated at the time of delivery. Results: Prior to the intervention, 3 out of 50 (6%) women engaged in mild–moderate physical activity for 150 min per week, while 20 out of 50 (40%) women participated in mild–moderate activity for 15–30 min, twice a week. Following the intervention, these percentages increased to 10 out of 50 (20%) and 31 out of 50 (62%), respectively (*p* < 0.05). Conclusions: This pilot study suggests that a single exercise session supervised by medical personnel may significantly improve the low physical activity levels observed in pregnant women.

## 1. Introduction

Regular physical activity has multiple beneficial effects on the general population, leading to improved physical and mental health [1,2]. However, only 50 percent of adults meet guidelines for aerobic physical activity, and approximately 80 percent of adults are not meeting guidelines for both aerobic and muscle-strengthening activity [3]. In pregnancy, regular physical activity is associated with numerous beneficial effects on the mother and the fetus [4], including a reduced frequency of excessive gestational weight gain, gestational diabetes mellitus (GDM), and postpartum weight retention [5,6]. The vast majority of international obstetrics and gynecology organizations recommend that, in the absence of contraindications, pregnant women should continue or commence physical exercise [7,8]. More specifically, they recommend 150 min of moderate-intensity physical activity per week. However, according to a population-based survey in the United States, 60 percent of pregnant women do not engage in physical activity, and according to another study, fewer than 15 percent of pregnant women met the generally accepted recommendation of engaging in 150 min of moderate-intensity physical activity per week [9,10].

Moreover, physical activity is the first line treatment in patients with gestational diabetes mellitus (GDM), as it improves glycemic control by reducing both fasting and postprandial blood glucose concentrations, leading to improved pregnancy outcomes [11,12,13]. However, even in pregnant women with GDM, the levels of physical activity are low [13].

The reasons explaining why pregnant women are reluctant to perform physical exercise during pregnancy vary widely, and many different interventions have been tested in order to tackle them and increase the levels of physical activity in these women [13,14,15].

The objective of this study is to investigate the impact of a single supervised physical exercise session on the overall physical activity levels of pregnant women with uncomplicated pregnancies and pregnant women with GDM.

## 2. Materials and Methods

### 2.1. Study Design

Pregnant women seeking consultations between February 2020 and February 2021 at the outpatient obstetric clinic of the Second Department of Obstetrics and Gynecology of Aristotle University in Thessaloniki were recruited and followed prospectively until the delivery of their offspring.

### 2.2. Participants

Pregnant women in the third trimester of pregnancy without any complications or with GDM were asked to participate in the study. The diagnosis of GDM was based on the results of a 75 g two-hour oral glucose tolerance test (OGTT), which takes place universally in all pregnant women in Greece between 24 and 28 weeks of gestation. The International Association of Diabetes and Pregnancy Study Groups (IADPSG) criteria (fasting blood glucose ≥ 92 mg/dL, 60 min blood glucose ≥ 180 mg/dL, or 120 min blood glucose ≥ 153 mg/dL) were used for the diagnosis of GDM [16]. Pregnant women with pre-existing diabetes mellitus (type 1 or 2), severe or moderate cardiovascular or respiratory disease, and pregnancies complicated by hypertensive disorders of pregnancy, ruptured membranes, persisting third trimester bleeding, placenta previa, cervical insufficiency, fetal growth restriction, and high-order pregnancy were excluded.

All women were thoroughly informed by the researchers about the safety and beneficial effects of physical exercise in pregnancy and were encouraged to engage in 150 min of moderate-intensity physical exercise per week, as per standard clinical practice in Greece. An informed consent form was signed by all of the study’s participants, and they consented to the use of their anonymized data for research purposes. The ethical committee and the institutional review board of the Medical School of Aristotle University of Thessaloniki approved the protocol of the study (protocol code 281, 27 February 2019). The study is in accordance with the Declaration of Helsinki.

### 2.3. Variables

Maternal and pregnancy characteristics were recorded, including maternal age, gravidity (number of times the woman has been pregnant), mode of conception, maternal height, pre-pregnancy weight and BMI, gestational age, and weight gain (Table 1).

The Greek version of the self-reporting International Physical Activity Questionnaire (IPAQ) was used to assess the physical activity status of the participants, and the IPAQ scores were estimated [17,18]. The IPAQ score was calculated based on the published formula, MET min per week: MET level x minutes of activity x events per week. Walking = 3.3 METs, Moderate Intensity = 4.0 METs, and Vigorous Intensity = 8.0 METs. Moreover, according to IPAQ, three levels of physical activity are suggested: (i) inactive, (ii) minimally active, and (iii) HEPA active (health-enhancing physical activity). In the inactive category are individuals who do not meet the criteria for the other two categories. In the minimally active category are individuals who meet one of the following criteria: (a) 3 or more days of vigorous activity of at least 20 min per day; (b) 5 or more days of moderate-intensity activity or walking of at least 30 min per day; or (c) 5 or more days of any combination of walking, moderate-intensity, or vigorous intensity activities achieving a minimum of at least 600 MET-min/week. In the HEPA active category are individuals who meet one of the following criteria: (a) vigorous-intensity activity on at least 3 days, achieving a minimum of at least 1500 MET-minutes/week; or (b) 7 or more days of any combination of walking, moderate-intensity, or vigorous intensity activities, achieving a minimum of at least 3000 MET-minutes/week.

In addition, the mothers’ blood pressure, heart rate, and blood glucose (in women with GDM) were measured and recorded. Subsequently, all participants underwent 30 min of moderate intensity (at 60–70% of the estimated maximum heart rate for their age) cycling on a stationary bicycle (KETTLER ERGO C4 Exercise Bike, Ense-Parsit, Germany). Before commencement of exercise, the bicycle’s seat was adjusted to the woman’s height (range of the height of the seat: 80–120 cm). Then, women were given 2 min to familiarize themselves with the bicycle, and once they were ready, they were asked to commence the exercise. In order to ensure moderate exercise intensity, all women used the heart rate monitor of the exercise bike during the aerobic exercise, and the rating of the perceived exertion scale ranged from 12 to 14 [19]. During the exercise, medical personnel encouraged the woman to keep up with verbal stimuli. In all participants, fetal ultrasonography was performed before, immediately after, and one hour after the exercise bout to assess the Doppler indices of the maternal uterine arteries (UtA), fetal umbilical artery (UmA), and fetal middle cerebral artery (MCA). In addition, venous blood sampling was carried out on all participants before the exercise bout, immediately after the exercise bout, and one hour after the exercise bout in order to determine their oxidation status. The methodology and the results of those analyses have been previously published [20]. At the time of delivery or the following 24 h, all women were asked to fill out the IPAQ again.

### 2.4. Statistical Methods

Continuous variables were presented as mean and standard deviation (SD), or as medians and interquartile range values. Categorical variables were summarized as percentages. Continuous variables were compared between the two time points (at the time of the exercise and at birth) using a paired *t*-test or Wilcoxon test. A t-test or Mann–Whitney test was used for the comparisons of the continuous variables between the groups of women with uncomplicated pregnancies and those with GDM. Chi-square or Fisher’s exact test were used for pairwise comparisons of proportions, and odds ratios (ORs) along with their 95% confidence intervals (CIs) were calculated. In all the above tests, a ***p***-value of <0.05 was considered significant. The analyses were carried out using the open-source software R 2.15.1 (The R Foundation for Statistical Computing).

## 3. Results

### 3.1. Participants

Two hundred and fifty pregnant women, attending consecutive appointments at the obstetric outpatient clinic of the Second Department of Obstetrics and Gynecology at Aristotle University in Thessaloniki for routine pregnancy check-ups, were initially evaluated for potential inclusion in the study. Out of this group, 32 pregnant women were identified as having gestational diabetes (GDM) and were invited to take part in the study; 25 out of these 32 agreed to participate. Following this, 30 women with uncomplicated pregnancies (out of the 250 consecutively examined) were carefully matched on a one-to-one basis with the previously enrolled 25 GDM pregnancies, considering factors such as pre-pregnancy BMI, maternal age, and gestational age. These 30 matched individuals were then approached to participate in the study, and ultimately, 25 of them consented to be part of the research. After the one-to-one matching in the two groups for the predefined characteristics, a statistical analysis was performed in order to assess the efficacy of our matching. Therefore, a total of 50 pregnant women consented to take part in the study (25 with GDM and 25 with uncomplicated pregnancies).

### 3.2. Descriptive Data

On the day of the exercise, the mean gestational age was 32.1 gestational weeks, the mean maternal age was 31.4 years, the mean BMI was 29.5, the mean BMI before the pregnancy was 29.5, the mean weight gain was 9.5 kg, and eight percent of the women had a history of GDM in a previous pregnancy. The characteristics of the overall population, along with the characteristics of pregnant women with uncomplicated pregnancies and those with GDM, are presented in Table 1. The only difference between uncomplicated pregnancies and pregnancies with GDM was the incidence of GDM in previous pregnancies (16% vs. 0%, *p* = 0.043).

### 3.3. Main Results

**Figure 1:**
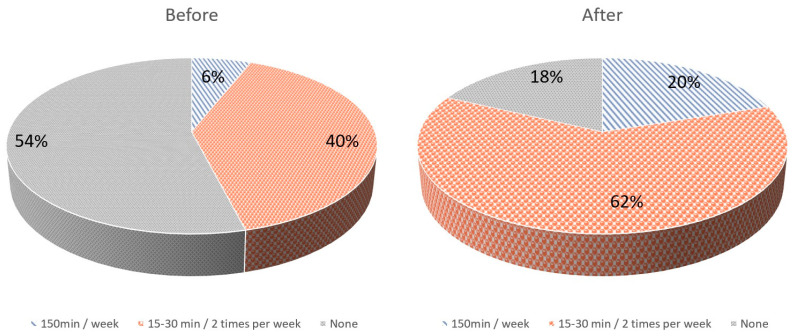
Physical activity levels of pregnant women before and after the intervention.

Before the intervention, 3/50 (6%) women reported undertaking some form of mild–moderate physical activity of 150 min per week, while 20/50 (40%) women reported undertaking some form of mild–moderate activity lasting 15–30 min twice a week. After the intervention, 10/50 (20%) women reported undertaking some form of mild–moderate physical activity of 150 min per week (*p* = 0.037, OR 3.91; 95% Confidence Interval 1.08–15.2), while 31/50 (62%) women reported having some form of mild–moderate activity of 15–30 min twice a week (*p* = 0.028, OR 2.44; 95% Confidence Interval 1.09–5.46) (Figure 1). Before the intervention, the IPAQ score was 108 ± 240 MET-min/week. After the intervention, the IPAQ score was 249 ± 311 MET-min/week (*p* < 0.001). Moreover, before the intervention, 3/50 (6%) were categorized as minimally active and 47/50 (94%) as inactive, according to IPAQ categorization. After the intervention, 10/50 (20%) were categorized as minimally active and 40/50 (80%) as inactive (*p* = 0.037).

**Figure 2:**
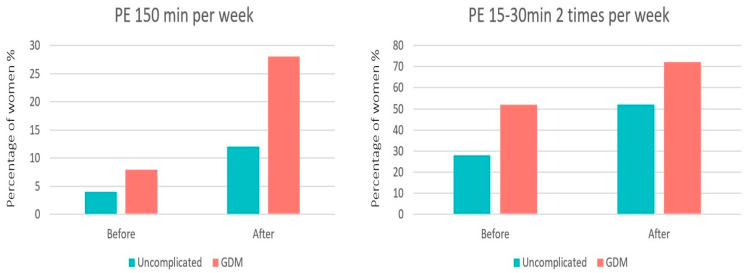
Physical exercise (PE) levels of pregnant women in the two groups, before and after the intervention.

In the uncomplicated pregnancies, before the intervention, 1/25 (4%) women reported having some form of mild–moderate physical activity of 150 min per week, while 7/25 (28%) women reported having some form of mild–moderate activity lasting 15–30 min twice a week. After the intervention, 3/25 (12%) women reported having some form of mild–moderate physical activity of 150 min per week (*p* = 0.291), while 13/25 (52%) women reported having some form of mild–moderate activity of 15–30 min twice a week (*p* = 0.083) (Figure 2).

In women with GDM, before the intervention, 2/25 (8%) reported having some form of mild–moderate physical activity of 150 min per week, while 13/25 (52%) of women reported having some form of mild–moderate activity lasting 15–30 min twice a week. After the intervention, 7/25 (28%) women reported having some form of mild–moderate physical activity of 150 min per week (*p* = 0.066), while 18/25 (72%) women reported having some form of mild–moderate activity of 15–30 min twice a week (*p* = 0.145) (Figure 2).

Comparing the proportion of pregnant women reporting having some form of mild–moderate physical activity of 150 min per week and the proportion of women reporting having some form of mild–moderate activity lasting 15–30 min twice a week between pregnant women with uncomplicated pregnancies and pregnant women with GDM, there were no statistically significant differences neither before the exercise bout nor after (at delivery) (*p* > 0.05).

## 4. Discussion

### 4.1. Main Findings

This study showed that for pregnant women in the third trimester of pregnancy, a single supervised exercise session could lead to an increase in their physical activity levels. More specifically, the supervised session of physical exercise increased the proportion of women who performed some form of mild–moderate physical activity of 150 min per week and the proportion of women performing some form of mild–moderate activity lasting 15–30 min twice a week. Moreover, IPAQ scores increased after the intervention.

### 4.2. Interpretation

Different interventions have been investigated in order to increase the levels of physical exercise among pregnant women, including educational sessions underlining the benefits of physical exercise, text and phone call reminders for physical exercise sessions, and mobile applications [21,22,23,24]. The results of those interventions are not homogenous. A recent systematic review on the topic showed that out of the 15 included studies, five showed an increase in the physical activity levels of pregnant women after the intervention [15]. However, none of the included studies used as an intervention a physical exercise session supervised by medical personnel. In this study, we showed that this type of intervention could incite pregnant women to increase their physical exercise levels.

Furthermore, in recent years, studies have attempted to identify the reasons why the majority of pregnant women do not meet the international recommendations regarding the levels of physical exercise during pregnancy. In one study, pregnant women stated that they do not believe it is possible to exercise during pregnancy, at least not without active support from antenatal care providers [14]. In another study investigating the same topic, some pregnant women stated that they believed that physical exercise during pregnancy was not safe or that they were uncertain about the safety of physical exercise in pregnancy [15]. The above-mentioned factors that negatively affect the levels of physical exercise in pregnancy were tackled by our intervention in the present study, as the medical personnel motivated and supervised pregnant women during the session of the physical exercise and reassured them about the safety of their fetuses by performing fetal sonography before, immediately after, and one hour after the session.

Moreover, the benefits of physical exercise in pregnant women with GDM are well established, leading to improved glucose management, primarily from increased tissue sensitivity to insulin, alleviating inflammation and oxidative stress, and improving endothelial function [20,25,26,27]. As a result, exercise can reduce both fasting and post-prandial blood glucose concentrations, and, in some patients with GDM, the need for insulin may be obviated. This is the reason why a program of moderate exercise is recommended as part of the treatment plan for patients with GDM, as long as they have no medical or obstetric contraindications to this level of physical activity [28]. Even though exercise is a therapeutic intervention in women with GDM, in the present study, only 8% reported having some form of mild–moderate physical activity of 150 min per week, while 52% of women reported having some form of mild–moderate activity lasting 15–30 min twice a week. However, our intervention applied in this study led to improved levels of exercise in those women. It is of high importance to show the different trends of the intervention in the different groups, GDM and uncomplicated pregnancies, as physical exercise serves a different role in each group, therapeutic and preventive, respectively.

### 4.3. Strengths and Limitations

This is the first study to assess the effect of a single supervised exercise session on the levels of physical activity of pregnant women. In addition, the present study assessed the engagement of pregnant women with GDM with physical exercise. A limitation of the study is that it was not designed to perform a subgroup analysis of the pregnancies, distinguishing uncomplicated and GDM pregnancies, thus being under-powered to perform such an analysis. This is probably the reason why there were no statistically significant changes within the subgroups. Moreover, an important limitation of this study is the absence of a control group of women who had not received the intervention. However, the already low levels of physical exercise during pregnancy tend to decrease further as pregnancy progresses, reaching their lowest point in the third trimester [29]. In our study, we successfully increased the levels of physical exercise during the third trimester. Therefore, we attribute this increase to our intervention, as the natural course of pregnancy would typically result in decreased levels of physical activity. The findings of this study are poised to pave the way for future research endeavors aimed at assessing the potential of this promising intervention.

## 5. Conclusions

The findings of this pilot study suggest that a single session of supervised exercise, followed by ultrasonography to demonstrate the safety of such exercise, could lead to increased physical activity in the third trimester of pregnancy. These findings should be addressed in future studies with appropriate controls and objective rather than self-reported measures of physical activity.

## Figures and Tables

**Table 1:** Characteristics of the included population of pregnant women.

Variable	All (*N* = 50)	GDM (*N* = 25)	Uncomplicated Pregnancies (*N* = 25)	*p*
Maternal age (years) Mean (SD)	31.4 (5.3)	32.4 (4.0)	30.4 (6.2)	0.222
Gestational age (weeks) Mean (SD)	32.1 (3.1)	32 (2.5)	31 (3.2)	0.186
BMI before pregnancy Mean (SD)	26.4 (6.4)	27.3 (7.9)	25.1 (5.2)	0.220
BMI during the study Mean (SD)	29.5 (5.6)	30 (5.7)	28.6 (5.0)	0.325
Weight gain (kg) Mean (SD)	9.5 (8.0)	8.2 (7.5)	10.7 (6.0)	0.261
Gravidity, *n* (%)				
I	32 (64%)	17 (68%)	15 (60%)	0.452
II	11 (22%)	5 (20%)	6 (24%)	
III	4 (8%)	2 (8%)	2 (8%)	
IV	2 (4%)	0	2 (8%)	
VIII	1 (2%)	1 (4%)	0	
History of GDM, *n* (%)	4 (8%)	4 (16%)	0	0.043
Spontaneous conception	50 (100%)	25 (100%)	25 (100%)	1.0

Quantitative variables are presented as means and standard deviations. Comparisons between the groups were carried out using the student’s *t*-test. Comparisons within the groups were carried out using a paired *t*-test. Qualitative data are presented in percentages. Comparisons between the groups were carried out using a Chi-square test.

## Data Availability

Data can be available after a reasonable request, describing thoroughly the proposal for their use, to the corresponding authors.
